# Three-Dimensional-Printed Sodium Alginate and k-Carrageenan-Based Scaffolds with Potential Biomedical Applications

**DOI:** 10.3390/polym16030305

**Published:** 2024-01-23

**Authors:** Cristina Stavarache, Sorina Alexandra Gȃrea, Andrada Serafim, Elena Olăreț, George Mihail Vlăsceanu, Maria Minodora Marin, Horia Iovu

**Affiliations:** 1 Advanced Polymer Materials Group, National University of Science and Technology POLITEHNICA București, 1-7 Gh. Polizu Street, 011061 Bucharest, Romania; cristina.stavarache@upb.ro (C.S.); sorina.garea@upb.ro (S.A.G.); andrada.serafim0810@upb.ro (A.S.); elena.olaret@upb.ro (E.O.); george.vlasceanu@upb.ro (G.M.V.); maria_minodora.marin@upb.ro (M.M.M.); 2 “C.D. Neniţescu” Institute of Organic and Supramolecular Chemistry, 202-B Splaiul Independentei, 060023 Bucharest, Romania; 3 Faculty of Medical Engineering, National University of Science and Technology POLITEHNICA Bucuresti, 1-7 Gh. Polizu Street, 011061 Bucharest, Romania; 4 Academy of Romanian Scientists, 54 Splaiul Independentei, 050094 Bucharest, Romania

**Keywords:** 3D printing, k-Carrageenan, sodium alginate, interpenetrated networks hydrogels scaffolds

## Abstract

This work reports the development of a marine-derived polysaccharide formulation based on k-Carrageenan and sodium alginate in order to produce a novel scaffold for engineering applications. The viscoelastic properties of the bicomponent inks were assessed via rheological tests prior to 3D printing. Compositions with different weight ratios between the two polymers, without any crosslinker, were subjected to 3D printing for the first time, to the best of our knowledge, and the fabrication parameters were optimized to ensure a controlled architecture. Crosslinking of the 3D-printed scaffolds was performed in the presence of a chloride mixture (CaCl_2_:KCl = 1:1; *v*/*v*) of different concentrations. The efficiency of the crosslinking protocol was evaluated in terms of swelling behavior and mechanical properties. The swelling behavior indicated a decrease in the swelling degree when the concentration of the crosslinking agent was increased. These results are consistent with the nanoindentation measurements and the results of the macro-scale tests. Moreover, morphology analysis was also used to determine the pore size of the samples upon freeze-drying and the uniformity and micro-architectural characteristics of the scaffolds. Overall, the registered results indicated that the bicomponent ink, Alg/kCG = 1:1 may exhibit potential for tissue-engineering applications.

## 1. Introduction

Due to its interdisciplinary nature, three-dimensional (3D) bioprinting technology has been progressively expanding in many fields, like polymer chemistry, pharmaceutical science, medicine, and biology. With the use of biopolymers, 3D printing shows great results in tissue engineering, medical devices, and drug-delivery systems. Furthermore, this technique is used for the fabrication of structures which mimic the cellular network, supporting cell adhesion and proliferation and thus the repair and development of new tissues and organs [1,2]. Three-dimensional printing is an additive manufacturing process in which the desired structure is assembled by extruding or binding materials in successive layers to obtain a 3D object [3]. The layer-by-layer process makes additive manufacturing a promising technology that can provide the most complex geometric scaffolds for delivering cells, genes and drugs into the human body using biopolymers [4,5]. Natural polymers like alginate, hyaluronic acid, chitosan, gelatin and agarose are some of the most widely used in hydrogel fabrication for tissue engineering and drug delivery due to their advantages like biocompatibility, non-toxicity, low cost, wide availability and ease of use compared with synthetic polymers [6,7,8]. However, biopolymer-derived hydrogels which are made from collagen and fibrin encounter some difficulties like weak mechanical strength [9]. Another problem for hydrogel-based bioinks is that after printing, the accuracy of the structure is limited and the capability of large-scale manufacturing is missing. Alginate and agarose have an advantage in bioprinting technology because of their high viscosity, indicating high resiliency during the printing process. Hence, adding them and other very viscous polymers into the bioink content can improve the printability for other more bioactive polymers like gelatin, fibroin, gelatin methacrylate that are less-printable inks [6]. To overcome these drawbacks, hydrogels-based bioinks are developed with shear thinning properties and rapid gelling in extrusion-based bioprinting in order to improve their mechanical strength and cytocompatibility and to increase cell viability, ensuring tissue growth and enabling printing of large-scale structures [10,11]. Therefore, biocomponent hydrogels, like interpenetrating networks, are seeing increasingly frequent use into the composition of bioinks because of their improved 3D-printing characteristics, including superior viscosities and shear-thinning. Due to their unique feature of combining the physical and chemical properties of manifold polymeric hydrogels in just one hydrogel, the interpenetrating networks hydrogels are gaining popularity in this industry [11].

Among various natural polymers that are used for bioink in biomedical applications, alginate and carrageenan are two commonly used polysaccharides because they can be crosslinked by a multitude of methods, they are not expensive sources of materials and they offer biocompatibility and no toxicity [7,12,13]. Alginate (Alg) is an anionic, linear polysaccharide extracted from brown algae. The alginate molecule is made of two monomers: α-L-guluronate (G) and β-D-mannuronate (M). The M-monomer units provide linearity and flexibility, while the G-monomer units are responsible for the rigidity of the polymer structure. Therefore, alginate that has elevated G-blocks content presents higher viscosity, and the G-monomers are in charge of the crosslinking process. Alginate used in tissue engineering and drug-delivery applications has the ability to form hydrogels in mild condition at room temperature because of crosslinkers like bivalent ions such as Ca^2+^ (the most frequently used is CaCl_2_) with G-unite and also due to the polyelectrolyte complexation with oppositely charged polymers [10,14,15,16]. Using alginate as bioink has its limitations when it comes to obtaining 3D-printing scaffolds with structural fidelity and integrity because of its weak shear modulus and the unstable crosslinking of the polymer. To improve alginate properties for printing, other biopolymers like modified chitosan and gelatin and also some additives like graphene oxide, carbon nanotubes, β-tricalcium phosphate hydroxyapatite and silica are added to the bioink’s composition [11,12,13]. Due to its hydrophilic character [10], alginate has minimal cell adhesion which can be improved by integrating gelatin and cells adhesion ligands to increase the viscosity and cytocompatibility of alginate bioink [13]. However, alginate hydrogel is a good candidate for tissue-engineering matrices because it allows transportation of the nutrient to the cells, it is capable of strengthening the cell walls and maintains a moist environment that preserves cells and drugs [17,18]. Also, alginate is a hemostatic agent and alginate fibers are used to produce dressing materials for slimy wounds; as a result, the scaffold made from these fibers are bioabsorbable and non-adherent [17]. All of these properties allow alginate and its multicomponent structures to be used in different fields such as pharmaceuticals and the biomedical industry [13].

Moreover, k-Carrageenan (kCG), is a linear, water-soluble, high-molecular-weight sulfated polysaccharide obtained from red algae, Rhodophyceae. Its molecule is composed of D-galactose residues linked alternately in three-linked-α-D-galactopyranose and four-linked-β-D-galactopyranose units [19,20]. Because of the negatively charged hydroxyl and sulfate groups in its backbone, carrageenan hinders inflammatory responses and ensures cell adhesion and proliferation [12]. Moreover, due to its excellent properties such as thickening, gelling, emulsifying and stabilizing agents, κCG is used in several fields like biomedical applications, food and the pharmaceutical industry. It is well known for its ability to form thermotropic and ionotropic gels [21] after cooling, even at room temperature and/or in the presence of mono- and divalent cations (K^+^, Na^+^, Ca^2+^), as a result of the transition from random coil, when heated, to a double-helix structure of polymeric chains of kCG [13,20,22] because of the hydrogen bonding [6,21]. Due to gelatin limitation, methacrylate-modified kCG and methacrylated gelatin were used by L. Tytgat [23] to obtain a biomaterial ink suitable for creating scaffolds that can be applied in adipose tissue engineering. Also, taking into account the different charged functional groups of gelatin and kCG, H. Li et al. [24] researched a divergent strategy for constructing the scaffold by printing these hydrogels alternately. The multilayered deposition is the result of the robust interfacial bonding caused by the electrostatic interaction between the polymers chains. The bioprinted kCG—gelatin structure showed an improved stability at 37 °C and great cell growth. Using nanosilicates together with k-CG, Wilson et al. [6], were able to obtain a rapidly gelling bioink with better printability and mechanical properties and it exhibited a high structural fidelity; therefore, complex physiologically suitable tissue were printed.

In a previous research work, Kim et al. [12], developed a printing material based on Alg and kCG using CaSO_4_ in the mixture. To overcome the use of the crosslinking agent incorporated in the ink, in this study, we optimized the mass ratio between both components (Alg and kCG) without the addition of the crosslinking agent inside the printing material. This was the first time this has been achieved, to the best of our knowledge. Moreover, we obtained suitable rheological properties of the Alg/kCG ink without the addition of crosslinking agent. The properties of the printed scaffolds were improved using different crosslinking concentrations of CaCl_2_—KCl mixture. This research presents the preliminary investigation for the development of a new 3D-printing biomaterial with possible application in tissue regeneration.

## 2. Materials and Methods

### 2.1. Materials

Average-molecular-weight sodium alginate (Alginic acid sodium salt from brown algae) and k-Carrageenan (predominantly κ and lesser amounts of λ carrageenan), potassium chloride (KCl) and potassium phosphate monobasic (KH_2_PO_4_) were purchased from Sigma-Aldrich, St. Louis, MO, USA; calcium chloride anhydrous powder (CaCl_2_) was purchased from Merck, Darmstadt, Germany, and sodium hydroxide pellets (NaOH) from Riedel-de Haën, Seelze, Germany. PBS solution was prepared in our laboratory.

### 2.2. Natural Polymers Based Inks Preparation

For the preparation of the inks, different amounts of kCG and Alg were added in distillated water to obtain the following weight ratios: Alg/kCG = 3:1; 1:1; 1:3, keeping the final concentration of the mixture at 2%. The polysaccharides were dissolved under magnetic stirring at 75–80 °C. These biopolymers mixtures were described in a previously reported study in which they were used to successfully obtain microparticles for protein drug-delivery systems [25] and drugs [26]. Moreover, kCG and Alg were used by M.H. Kim et al. [12] to optimize 3D-printing conditions in order to fabricate enhanced mechanical properties scaffold using Alg-CaSO_4_ and Alg-k-CG-CaSO_4_ hydrogels. In their study, CaSO_4_ was used as the crosslinking agent.

### 2.3. Rheological Characterization of Precursors

The rheological properties of biopolymers solutions were evaluated using a Kinexus Pro rheometer (Malvern Instruments, Brussels, Belgium) equipped with a Peltier element for precise temperature control. To assess the rheological properties of the materials, a parallel plate geometry was used. Following the protocol described in [12], the viscosity (η) of the biopolymers solutions was conducted in the shear rate interval 0.01–1000 s^−1^ at 25 °C. Frequency sweep measurements were recorded between 0.10–10 Hz to evaluate the storage modulus (G′) and the loss modulus (G″) at a constant shear stress of 1 Pa. A water lock was used during measurements to protect the solutions from drying. The recovery properties of the bicomponent solutions were measured in three steps in order to examine the rheological behaviors of the samples during the 3D-printing process: in the first step, a very low shear rate of 0.1 s^−1^ was applied for 60 s; in the second step, the deformation phase, the shear rate of 100 s^−1^ was tested for 10 s. In the third phase, the recovery step, was examined again at a very low shear rate of 0.1 s^−1^ for 300 s. Rheological studies were performed in triplicate.

### 2.4. Three-Dimensional Printing of the Inks

The prepared Alg/kCG formulations were printed using a four-head 3D Discovery Bioprinter from RegenHU Ltd., Villaz-St-Pierre, Switzerland and the layer-by-layer deposition technique was employed to obtain the 3D scaffolds using the Direct Dispenser DD135N print-head from RegenHU Ltd., Villaz-St-Pierre, Switzerland.

The printing materials were poured into a 3 mL cartridge, avoiding air bubbles, and attached to different cylindrical nozzles depending on the tested material: 23 gauge (ø 0.33 mm, needle length 6.35 mm), 25 gauge (ø 0.25 mm, needle length 6.35 mm) or 27 gauge (ø 0.20 mm, needle length 12.7 mm). Before the syringe was loaded, the biopolymers mixtures were heated to form a fluid phase and slowly poured into the cartridge and let it cool at room temperature. The printability of the three inks Alg/kCG = 3:1; 1:1; 1:3 was tested to ensure successful printing through a number of trials. Different pressures ranging from 20 to 180 kPa and feed rates ranging from 5 to 10 mm/s were employed in order to optimize these two important printing parameters. All the tests were carried out at room temperature on glass microscope slides and the design of the scaffold was manufactured with the help of the BioCAD 1.1 software. After the trials, only the Alg/kCG = 1:1 ink was found to be a proper candidate for printing. Therefore, the 3D scaffolds of Alg/kCG = 1:1 ink exhibiting a circle grid-like structure were printed through the 27 gauge with an inner diameter of 0.20 mm at several printing speeds (6, 8, 10 mm/s) and extrusion pressures ranging from 100 to 180 kPa. The cylindrical grid 3D-bioprinted scaffolds structure consists of various layers: 10, 20, 40 and 60 layers with a 0–90° deposition direction, line space: 2, 5; 3; 4 and 5 mm; the diameter of the circle was 15 and 20 mm.

**Figure 1:**
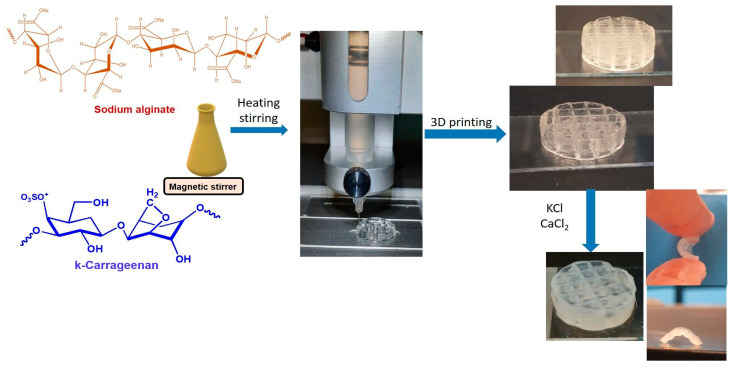
Schematic steps representation of the printing process, including synthesis of the bicomponent material and the crosslinking of the manufactured 3D-printed scaffold Alg/kCG = 1:1.

After manufacturing, the 3D depositions of Alg/kCG = 1:1 ink were immersed in a crosslinking solution of different concentrations of CaCl_2_:KCl = 1:1 (volumetric ratio) of 0.3 M (noted M1), 0.5 M (noted M2), 0.8 M (noted M3) and 1.2 M (noted M4) for 30 min at room temperature, allowing the Ca^+2^ and K^+^ ions to harden the structures. The objects were rinsed with deionized water. Figure 1 illustrates the synthesis of the bicomponent ink, the printing process and the Alg/kCG = 1:1 scaffold obtained before and after crosslinking.

Additionally, to investigate the mechanical properties of the crosslinked hydrogels, membranes of each composition were obtained by following a similar procedure. The previously described formulations and control samples were poured in silicon molds with a diameter of 20 mm and a height of 0.75 mm and immersed in the crosslinking solution at room temperature for approximately 30 min. Subsequently, the samples were washed with distilled water and tested.

### 2.5. Swelling Behavior of the 3D-Printed Hydrogel Structures

The swelling behavior of the 3D-printed structures was conducted in PBS pH = 7.4 at physiological temperature (37 °C), using a thermostat water bath GFL 1083, Kischer Biotech, Steinfurt, Germany. The swelled scaffolds were removed at predetermined time intervals and weighed after being blotted off using filter paper to remove the remaining residual liquid.

The swelling degree (SD, %) was calculated using the following Equation (1) [27,28]:

(1) $$SD,\%=\frac{\mathrm{Wt}\;-\;W0}{W0}\times 100,$$

where W0 is the initial dried weight of the scaffold (before being submerged in PBS) and Wt is the mass of swollen scaffold at the predefined sampling points, t.

At swelling equilibrium, the maximum swelling degree (MSD, %) was evaluated using Equation (2) [27,29].

(2) $$MSD,\%=\frac{\mathrm{WE}-W0}{W0}\times \;100,$$

where WE is the equilibrium weight of the sample and W0 is the initial dried mass of the scaffold.

The experiment was performed in triplicate and the average values were reported.

### 2.6. Structural Stability and Degradation of 3D-Printed Scaffolds

The structural stability of the 3D-printed scaffolds was determined in a thermostat water bath GFL 1083, Kischer Biotech, Steinfurt, Germany by individually submerging the dried samples in PBS, pH = 7.4 at physiological temperature (37 °C). Preliminary construct degradation degree (DD) was calculated using Equation (3).

(3) $$DD,\%=\frac{\mathrm{Wf}\;-\;\mathrm{Wmax}}{\mathrm{Wmax}}\times 100$$

where Wmax represents the maximum weight and Wf is the remaining weight value of the swollen sample on different days. The 3D-printed objects were dabbed and weighted. The experiment for evaluating the structure stability of the 3D-printed scaffolds was performed using 3 independent samples for each group.

### 2.7. Mechanical Properties of the Hydrogel

The obtained hydrogels’ response to an applied effort has been determined at both macro- and micro-scale, through rheology and nano-indentation tests, respectively. The membranes obtained through the crosslinking of the synthesized inks were used.

The rheological behavior of Alg/kCG = 1:1 bicomponent hydrogel resulting from crosslinking with different concentrations of KCl:CaCl_2_ = 1:1 (volumetric ratio) was studied via dynamic oscillation measurements at 25 °C using a Kinexus Pro rheometer. A normal force of 0.5 N was maintained during the measurements. Also, a water lock was used in order to prevent dehydration of the hydrogels. Dynamic oscillation tests were measured in the frequency range 0.10–10 Hz.

Dynamic instrumented indentation tests. Surface mechanical properties were investigated using a G200 Nano Indenter system (KLA Instruments, Milpitas, CA, USA) equipped with a DCM II head and CSM option. The storage modulus (G′) and loss modulus (G″) were computed according to the “G-Series DCM CSM Flat Punch Complex Modulus, Gel” method implemented within NanoSuite 6.52.0 software. Indentation tests were performed along samples’ filaments using a cylindrical diamond tip with a flat-ended punch of 100 µm diameter. Five indentations (7 µm in depth) at 10 Hz testing frequency and 500 nm oscillation amplitude were performed on each sample, ensuring 500 µm distance between them. The results were reported as mean ± standard deviation (*n* = 5) for a Poisson’s ratio value of 0.4. The samples were analyzed when they were freshly printed and crosslinked with different molar concentrations of 1:1 volumetric ratio of ionic solutions of CaCl_2_ and KCl.

### 2.8. Morphology Analysis

For the micro-computer tomography analysis of the crosslinked 3D-printed scaffolds, a SkyScan µCT 1272 high-resolution equipment (Bruker microCT, Kontich, Belgium) was employed. For the morphostructural characterization of the 3D-printed hydrogels, the dried samples were placed on the scanning stage and fixed in place with dental wax. The scanning was performed at room temperature by rotating the object in front of the source (voltage 50 kV, current 130 mA) for 190 degrees with a rotation step of 0.2 degrees, with each frame resulting from averaging 3 projections per frame (300 ms/frame). The scanning resolution (image pixel size) was set at 12 µm for all printed samples. Tomograms were reconstructed from the raw data in Bruker NRecon 1.7.1.6 software. Bruker CTAn 1.17.7.2 software was employed to analyze the tomograms and measure the morphological parameters of the printed objects (total porosity, pore/wall size distribution, etc.) and to generate the secondary color-coded dataset depicting wall thickness variations. All procedures were performed after thresholding (binarization—white pixels for solid sample, black pixels for pores) and despeckling (removal or residual scanning artifacts) and were based on the image pixel size for conversion into metric units.

## 3. Results and Discussion

### 3.1. Rheological Characterization of the Precursors

**Figure 2:**
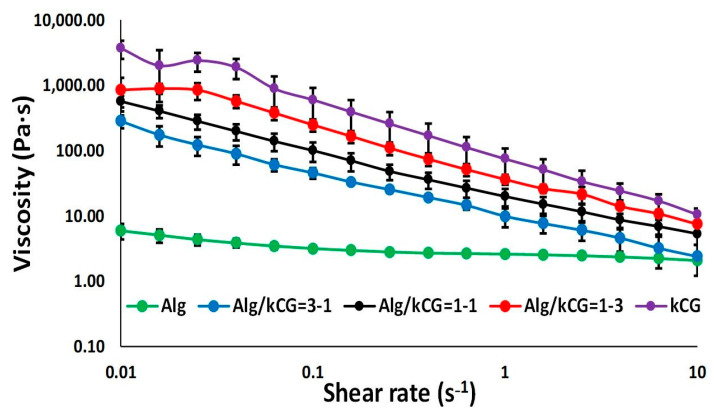
The viscosity dependence on the shear rate at 25 °C.

Rheological studies were performed on all three inks (Alg/kCG = 1:3; 1:1 and 3:1). Alg and kCG solutions with a concentration of 2% were also subjected to rheological measurements as control. Shear viscosity data obtained at 25 °C for all samples are plotted in Figure 2.

All solutions show shear-thinning behavior, as their viscosity decreases with the increase in shear rate. The Alg solution presents an extended Newtonian plateau, with its viscosity starting to decrease at significantly higher shear rate values compared to the bicomponent formulations. Conversely, kCG solution has the highest viscosity due to the biopolymer’s double-helix structure, which leads to the formation of a gel-like composition at low temperature (25 °C) [30].

**Figure 3:**
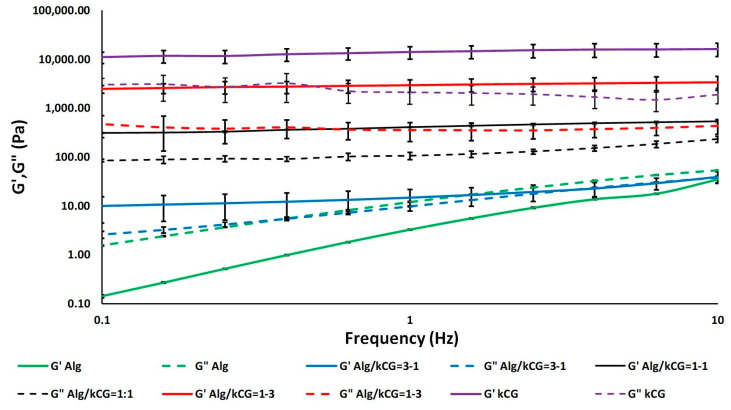
The dependence of storage modulus G′ and loss modulus G″ on frequency for the studied solutions.

As depicted in Figure 2, the curves registered for the bicomponent formulations are placed between those of the control solutions, indicating that the presence of kCG increases the viscosity of the inks. This behavior is attributed to the formation of hydrogen bonds between the –OH groups of the two biopolymers [31]. However, at an Alg/kCG ratio of 1:1, the inks are more viscous and more homogenous when compared with the other bicomponent formulations, as depicted in Figure 3. The frequency sweeps performed in the range 0.1–10 Hz offer information on the inks’ stability. The results are presented as logarithmic graphs in Figure 3.

The Alg control and Alg/kCG = 3:1 samples exhibit significantly different behavior when compared to the kCG and the other two bicomponent formulations. The samples in which the alginate is the predominant component exhibit a liquid-like behavior, with G″ dominant over the G′. Furthermore, these two compositions are highly influenced by the frequency and display an increase in G′ and G″ with the increase in the frequency.

Increasing the kCG content to an Alg/kCG weight ratio of 1:1 and 1:3 leads to formulations with a gel-like behavior and higher stability in the studied frequency range.

**Figure 4:**
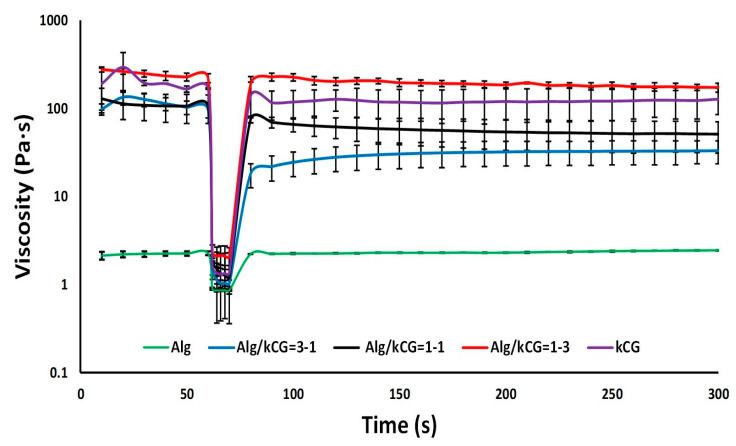
The recovery behaviors of the bicomponent formulations subjected to alteration of shear rates (0.1 s^−1^ and 100 s^−1^) at 25 °C.

The thixotropic behavior of the bicomponent formulations was examined in order to understand how their structures react to high-speed shearing. During printing, the structure of the ink might be broken due to the high shear rate. The recovery behavior experiment is performed in the first step at a low shear rate of 0.1 s^−1^ for 60 s to simulate the stationary state of the ink before extrusion, as depicted in Figure 4, followed by a second step in which a high shear rate of 100 s^−1^ was applied for 10 s to simulate the condition of the ink during extrusion through the nozzle. In the third phase, the same parameters as phase 1, only for a longer time, were used in order to mimic the state of the sheared ink after printing, and structure recovery was detected [12,32]. The results from the creep and recovery evaluation are presented in Figure 4. As shown, all solutions’ viscosity decreased significantly at high shear rate and recovered rapidly at low shear rate except for the Alg/kCG = 3-1 bicomponent ink, whose structure was probably broken by the shear rate conditions, and no full recovery (35%) was observed until the end of the trail. For the Alg/kCG = 1-1 sample, the obtained results indicated a recovery time of 10 s with a 58% recovery of the initial viscosity. Its viscosity degreased from 129.23 Pa∙s at s^−1^ to 1.20 Pa∙s at 100 s^−1^ and then, during a 10 s period, rapidly increased to 75.27 Pa∙s. In the case of Alg/kCG = 1-3 formulation, the recovery percentage was 82% in 20 s and even if more time was provided, it would not boost any further. Moreover, the recovery time for Alg and kCG was 10 s. Because of the samples’ rapid and reversible viscosity response, demonstrating reversible structure transition, they can be used for 3D printing, since they can be easily extruded while rapidly recovering enough mechanical strength to support the following extruded layer [33].

### 3.2. Three-Dimensional Printing of the Alg/kCG Formulations

Printing a 3D scaffold from a hydrogel is quite challenging. The content of the water is very high in a biomaterial, which makes it a soft material, and the mechanical strength of the hydrogel structure is not strong enough to sustain the weight of the whole pattern. Therefore, the mechanical strength and viscosity of the hydrogel material employed has to be sufficiently high to support the pressure of the scaffold construction weight [33]. Also, the ink must flow continuously and uniformly upon extrusion through a needle. The ink obtained from the mixture of the two polysaccharides, kCG and Alg, was studied in order to obtain a suitable ink for a 3D-printing construct.

To manufacture the printed objects, extrusion-based 3D printing was used and a layer-by-layer deposition technique of the inks was engaged. The ability of the inks to be printed, to form a continuous filament, as well as the printing parameters, like writing velocity and extrusion pressure, were initially tested on a simple, 1D-printed shape, a circle with a grid. To evaluate the structural strength of the object, a 3D scaffold with an upper layer was printed.

The solution of 2% kCG could not be printed using the print-head at room temperature because of the formation of a solid-gel structure of the kCG, due to the hydrogen bonds between the polymeric chains [6] which increase with the increase in kCG concentration. In order to break this hydrogen bonding, for the extrusion, the ink must be heated.

**Figure 5:**
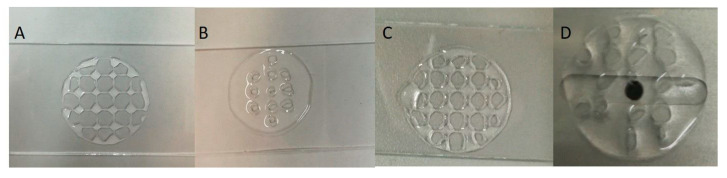
Printed structures using Alg/kCG = 3:1. All objects have 5 layers with 4 mm line spacing and were printed at 10 mm/s velocity using a 27 G needle at different extruding pressures like 40 kPa (**A**); 100 kPa (**B**) and 50 kPa (**C**) and a 23 G needle at a 25 kPa pressure (**D**).

**Figure 6:**
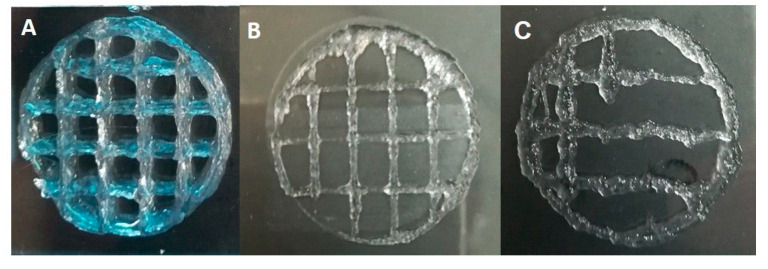
Printed structures using Alg/kCG = 1:3. All trails were performed using a G 27 nozzle. The structures (**A**,**B**) were extruded at 150 kPa with 10 mm/s for (**A**) and 8 mm/s for (**B**,**C**). The pressure used for manufacturing C construct was 180 kPa.

Multiple printability tests were performed to determine the optimal printing parameters. In the case of the bicomponent inks, Alg/kCG = 3:1 and Alg/kCG= 1:3, using 23-gauge or 27-gauge needles with different writing speeds and pressure had the result of an unequal scaffold and also structural collapse. The results are illustrated in Figure 5 and Figure 6.

The thick filaments produced by Alg/kCG = 1:1 ink using the G27 nozzle were continuous, homogeneous and the deposition was subjected to several printing parameters to evaluate the structural fidelity. The Alg/kCG = 1:1 ink has the ability to print constructs that support multiple layers without collapsing. The parameters used to print the scaffolds were 8 mm/s ± 2 for printing speed and 115, 120, 140 kPa and 180 kPa for extrusion pressure. The printing parameters optimization is presented in Table 1.

The shape fidelity and repeatability were assessed via 3D printing of six scaffolds using the same manufacturing characteristics, 8 mm/s for printing speed and 115 kPa for extrusion pressure.

**Figure 7:**
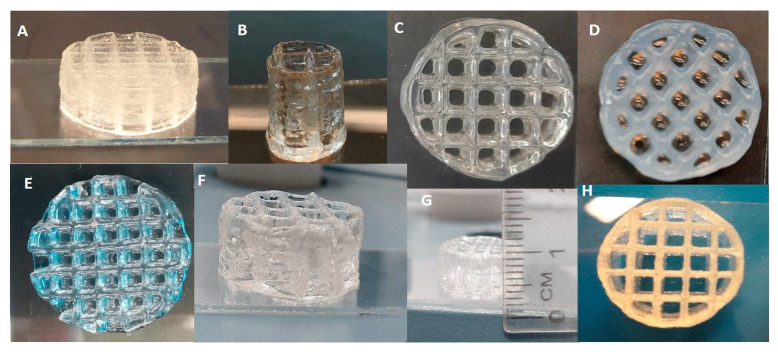
Three-dimensional layer-by-layer depositions of Alg/kCG = 1:1 ink. Macroscopic imagine (**A**) represent a 35-layer scaffold; imagine (**B**) displays a 60 layers construct; imagine (**C**) shows a 20-layer scaffold prior crosslinking while (**D**) shows a 20-layer scaffold after crosslinking. A 15-layer grid construct is presented in (**E**). The imagines noted with (**F**,**G**) depict the side view of a 40-layers scaffold. The imagine (**H**), illustrates the top view of a 35-layer scaffold.

After printing, the 3D scaffolds obtained with Alg/kCG = 1:1 ink were ionic crosslinked for 30 min using Ca^+2^ and K^+^ to enhance the mechanical properties of the structures. Prior to this crosslinking procedure, the printed sample had good stability because of the thermo-reversible gelation of kCG, a unique feature of this polysaccharide, and could support itself [6,34,35]. The reinforcement of the scaffolds was achieved with the crosslinking of the two biopolymers through the synthesis of the interpenetrating ionic network between kCG and Alg in the presence of Ca^+2^ and K^+^. The crosslinking of the kCG leads to the formation of the ordered double-helix structure of the chains due to the ionic interaction between the monovalent and divalent ions and the sulfate and hydroxyl group [34,36] and the stability of the Alg hydrogel is achieved via the ionic interaction of the Ca^+2^ ions with the –COO^−^ group of the L-guluronic acid of the Alg chain [34,37] and, additionally, because of the formation of hydrogen bonds between the two polymers [31]. Multiple 3D structures with different geometries are illustrated in Figure 7.

A 35-layer grid cylinder with 4 mm line space printed using an 8 mm/s printing speed and 180 kPa is displayed in Figure 7A,H. Complex objects were made to illustrate the shape restraint under the weight of multiple layers (40 layers; 60 layers, illustrated in Figure 7F and Figure 7B, respectively). The 40-layer lattice network side view is shown in Figure 7G. Also in Figure 7C,D is a 20-layer scaffold prior to (C) and after crosslinking (D) manufactured using an 8 mm/s printing speed and 120 kPa pressure. A 15-layer grid construct with 4 mm line space that was made at 140 kPa with a 10 mm/s feed rate is depicted in Figure 7E.

### 3.3. Mechanical Characterization of the Obtained Hydrogel

**Figure 8:**
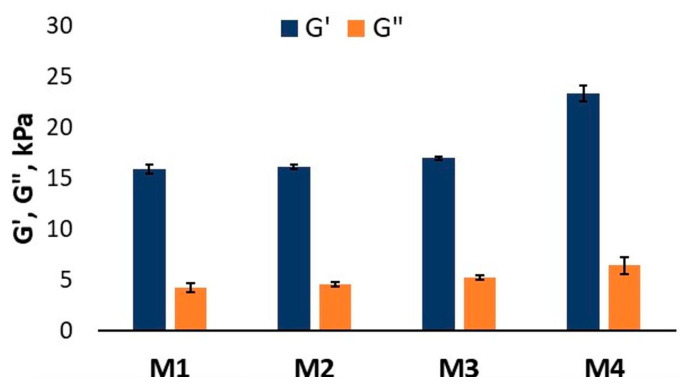
Graphical representation of storage modulus and loss modulus as obtained from indentation tests.

The effect of different concentrations of the crosslinking solutions on the mechanical properties at micro-scale was evaluated through indentation tests (Figure 8). G′ and G″ were obtained for freshly printed and crosslinked samples, as previously described [38]. The results were found to be similar between samples crosslinked with M1 (G′ = 15.93 ± 0.93 kPa, G″ = 4.23 ± 0.42 kPa), M2 (G′ = 16.18 ± 1.02 kPa, G″ = 4.54 ± 0.23 kPa) or M3 (G′ = 17.00 ± 1.46 kPa, G″ = 5.26 ± 0.19 kPa). However, 46.84%, 44.57% and 37.60% increases in storage modulus values were obtained for samples crosslinked with M4 (G′ = 23.39 ± 1.29 kPa, G″ = 6.45 ± 0.82 kPa) when compared to M1, M2 and M3, respectively.

The results obtained from nanoindantation measurements are in agreement with the result from macro-scale tests suggesting a homogeneous crosslinking at the surface as well as within the whole object for all samples.

### 3.4. Swelling Behavior of the 3D-Printed Hydrogel Structure

One of the main characteristics of the hydrogels used in the medical field is represented by water uptake capacity and their behavior when coming into contact with simulated biological fluids [38].

**Figure 9:**
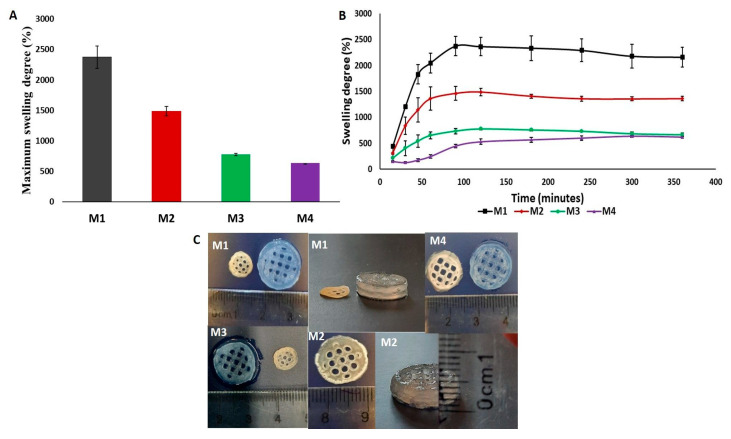
(**A**) Maximum swelling degree of the crosslinked scaffolds in PBS, pH = 7.4; (**B**) Swelling kinetics of the 3D-printed constructs; (**C**) Images of 3D-printed specimens during swelling trials showing the 3D construct before (left) and after (right) the sinking in PBS, pH = 7.4 and the side view of the scaffolds.

The diffusion of water molecules into the 3D-printed construct was observed through the evaluation of the swelling rate of the dried and crosslinked scaffolds at different concentrations in PBS, pH = 7.4 at 37 °C, and it is displayed in Figure 9A.

From Figure 9A,B, it can be seen that, in this case, the SD and MSD is only governed by the increase in crosslinking concentration. The swelling results for the construct that was crosslinked with the highest molar concentration showed that it reached its swelling equilibrium value after 5 h and it had the lowest MSD of the four studied concentrations. The equilibrium point for the Alg/kCG = 1:1 crosslinked with M2 and M3 was obtained in 2 h and for the scaffolds that were crosslinked with M1, the equilibrium swelling degree was obtained in 90 min. As the concentration of the crosslinking agent increased, the swelling rate reduced and the swelling equilibrium time was longer because of the enhancement of “egg-box” gelation mechanism [39] from Alg and Ca^+2^ and a stronger gelation from the kCG part through coil-to-helix transition with K^+^ and Ca^+2^ and a three-dimensional network of double helixes of polymeric chains with the same cations [8,40]. The results of the swelling tests were in agreement with the observed behavior from mechanical testing at micro-scale; the samples crosslinked with M4, with the highest storage modulus (G′ = 23.39 kPa), recorded the lowest maximum swelling degree (MSD = 634.4%) during a longer period of time (5 h), while samples crosslinked with M1 with the lowest storage modulus (G′ = 15.93 kPa) recorded the highest MSD (MSD = 2375.6%) in a shorter time (90 min).

During rehydration, all studied 3D scaffolds regained their shape, as is illustrated in Figure 9C.

### 3.5. Structure Stability and Degradation of 3D-Printed Scaffolds

**Figure 10:**
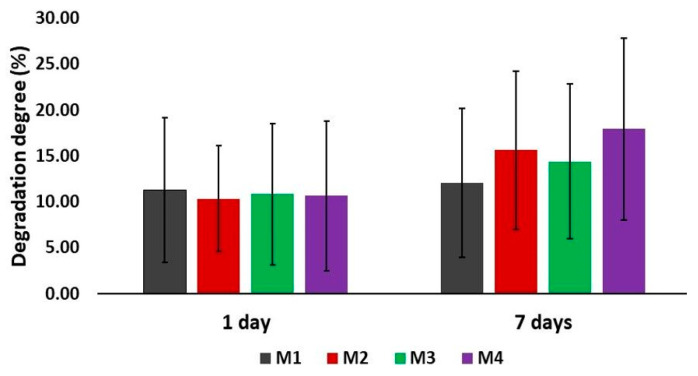
Degradation analyses of the bi-component Alg/kCG = 1:1 3D-printed scaffolds.

The stability and degradation tests on the dried 3D-printed Alg/kCG = 1:1 scaffolds were also studied in PBS, pH = 7.4 at 37 °C during several days, and the results are depicted in Figure 10.

Hydrolysis is the process commonly used to mediate degradation, which can happen in the matrix of the polymer or at the crosslinks. The weight loss was not significantly influenced by the increase in the crosslinkers’ concentration. After 24 h, all scaffold presented almost the same mass loss with DD% around 10% to 11%. This tendency was also observed after 7 days; no significant difference in the degradation degree was noticed for all the tested scaffolds (DD% = 12% for 0.3 M, DD% = 16% for 0.5 M, DD% = 14% and DD% = 18% for 0.8 M). The samples crosslinked with M1, M2 and M3 had close values of mass loss; the ones hardened with M4 showed the highest percentage of degradation rate.

### 3.6. Morphology Analysis

Micro-CT analysis enabled the evaluation of the prints’ morphology (printing fidelity, porosity and ink structure upon freeze-drying) in a qualitative and quantitative manner. Figure 10 depicts the reconstructed tomograms of the four samples. The square lattice model is preserved. The color-coded rendering of the tomograms is associated with the thickness of the solid walls formed during the pore-inducing process. According to this, as the concentration of crosslinker increases, thicker walls emerge.

**Figure 11:**
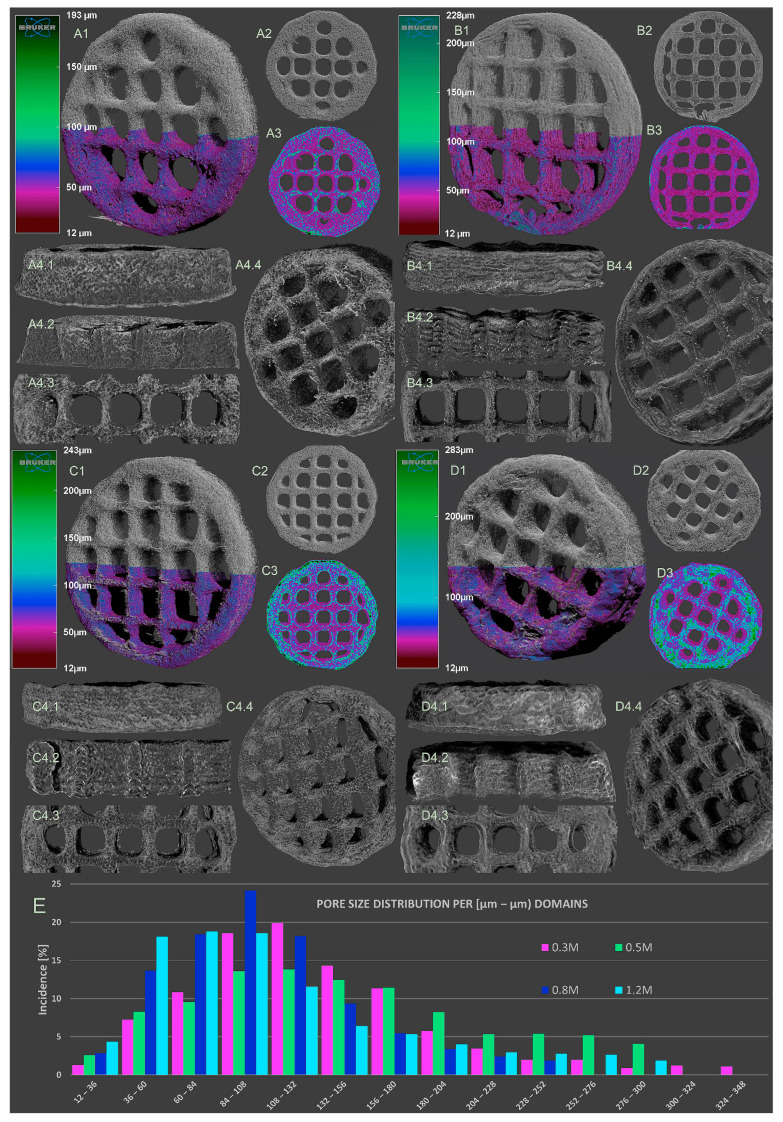
Micro-CT images depicting the surface of whole (**A1**) 0.3 M, (**B1**) 0.5 M, (**C1**) 0.8 M and (**D1**) 1.2 M consisting of original tomograms (grayscale half) overlapped with CTAn-processed color-coded dataset (bottom half). Secondary and ternary subsets depict the same top-view section for each printed object providing the image of pore/wall interface (**A2**–**D2**) and the solidity of inner solid structures (**A3**–**D3**). The “4” subsections depict the original tomogram of the objects with the focus on the outer aspect of the prints, as follows: 4.1—lateral view, 4.2—cross-section to illustrate the aspect of the external walls of the grid, 4.3—top view of the central areas of the samples and 4.4—overall surface aspect of the printed objects. (**E**) division covers the plot of the quantitative analysis of pore size within the scanned samples).

To begin with, the outer regions, better illustrated in subsections A1–A4 of Figure 11, are characterized by homogenous walls which usually extend up to 100 µm. The outer aspect of the printed objects features uniform open pores, which are essential to facilitate the exchange of nutrients and metabolites with the innermost domains. The narrow size range of walls and external pores indicates a balanced patterning of these key features. Homogeneity could be one of the factors that support the stability of the object, since there is no disparity between the fine walls and the pore network. Also, the process of freeze-drying did not promote the formation of unusually large cavities that could weaken the object. In the cross-sectional views (B1–B4), the dense arrangement of walls defines a pore system with a seemingly consistent size distribution; this observation is also supported by the measurement that aimed to assess their variability within the printed mesh (excluding the square-shaped macropores of the CAD model) (Table 2).

In contrast, the inner walls from the filaments are thicker and are stimulated to arrange in a more compact form as the gelling agent is added in larger amounts. This trend is highlighted in subsection C in Figure 11, in which the slices show more turquoise and green domains far away from the periphery. This could be attributed to the fact that increasing the crosslinking concentration leads to the formation of smaller pores (probably an important share under the detection limit of the scanning) during the freezing process.

Generally, the pore size distributions respect a Gaussian bell (Figure 10) in all printed formulations, but their span reduces with the increase in the content of the CaCl_2_ and KCl solution. In consequence, the profiles of denser crosslinked inks are becoming more and more left-skewed, since the rheology of the precursors constrained the pores to expand freely.

## 4. Conclusions

New formulations using Alg and kCG in different ratios were obtained, and their printability was tested. Of the tested biomaterials, the Alg/kCG = 1:1 ink presented the best printability and the constructs created with this ink maintained their shape without collapsing. The swelling behavior of the 3D-printed scaffold was influenced by the increasing ionic concentration. The lowest swelling degree was observed for the samples crosslinked with the highest concentration, which also presented the highest storage modulus and viscous modulus on the tests performed to assess mechanical properties. Over a period of 7 days, the tested samples crosslinked with M4 showed the highest percentage of degradation rate. Micro-CT analysis completed on freeze-dried scaffolds exhibited printing fidelity, porosity, and homogenous structure of all printed depositions. As a result, Alg/kCG = 1:1 ink provided better printability and printing fidelity and can be considered a suitable option in the development of 3D-printed scaffolds for tissue engineering.

## Figures and Tables

**Table 1:** Printing parameters used to print the polysaccharides inks.

Material	Parameters		
	Extrusion Pressures (kPa)	Printing Speed (mm/s)	Gauge Needles
Alg/kCG = 1:1	115–140	6, 8, 10	27
Alg/kCG = 1:3	150–180	8, 10	27
Alg/kCG = 3:1	25, 30, 40–100	10	23, 25, 27

**Table 2:** Total porosity and specific surface (as ratio of object surface/object volume).

Sample	Parameters	
	Obj s/vol (μm^−1^)	Porosity (%)
M1	66.6 × 10^−2^	56.0
M2	7.29 × 10^−2^	61.2
M3	6.81 × 10^−2^	41.8
M4	3.82 × 10^−2^	33.5

## Data Availability

Data are contained within the article.
